# The Importance of Ubiquitination and Deubiquitination in Cellular Reprogramming

**DOI:** 10.1155/2016/6705927

**Published:** 2016-01-06

**Authors:** Bharathi Suresh, Junwon Lee, Kye-Seong Kim, Suresh Ramakrishna

**Affiliations:** ^1^Graduate School of Biomedical Science and Engineering, Hanyang University, Seoul 133-791, Republic of Korea; ^2^Department of Physiology and Brain Korea 21 PLUS Project for Medical Science, Yonsei University College of Medicine, Seoul 120-752, Republic of Korea; ^3^College of Medicine, Hanyang University, Seoul 133-791, Republic of Korea

## Abstract

Ubiquitination of core stem cell transcription factors can directly affect stem cell maintenance and differentiation. Ubiquitination and deubiquitination must occur in a timely and well-coordinated manner to regulate the protein turnover of several stemness related proteins, resulting in optimal embryonic stem cell maintenance and differentiation. There are two switches: an E3 ubiquitin ligase enzyme that tags ubiquitin molecules to the target proteins for proteolysis and a second enzyme, the deubiquitinating enzyme (DUBs), that performs the opposite action, thereby preventing proteolysis. In order to maintain stemness and to allow for efficient differentiation, both ubiquitination and deubiquitination molecular switches must operate properly in a balanced manner. In this review, we have summarized the importance of the ubiquitination of core stem cell transcription factors, such as Oct3/4, c-Myc, Sox2, Klf4, Nanog, and LIN28, during cellular reprogramming. Furthermore, we emphasize the role of DUBs in regulating core stem cell transcriptional factors and their function in stem cell maintenance and differentiation. We also discuss the possibility of using DUBs, along with core transcription factors, to efficiently generate induced pluripotent stem cells. Our review provides a relatively new understanding regarding the importance of ubiquitination/deubiquitination of stem cell transcription factors for efficient cellular reprogramming.

## 1. Introduction

Pluripotent stem cells, which are derived from the inner cell mass (ICM) of the blastocyst, are characterized by unlimited self-renewal and they can be triggered to differentiate into all three embryonic germ layers: (i) ectoderm, skin and nerve; (ii) mesoderm, bone, blood, and muscle; and (iii) endoderm, gut and lung tissues. In 1998, the first human embryonic stem cells (hESCs) derived from the ICM of a preimplantation blastocyst were isolated [1]. Thereafter, several human ES cell lines became available to researchers for the generation of cells of multiple lineages [2]. Thus, the capacity to culture embryonic stem cells and induce them into different cell types under defined* in vitro* conditions has revolutionized developmental biology [3].

## 2. Induced Pluripotent Stem Cells

Induced pluripotent stem cells are defined as differentiated cells that have been experimentally reprogrammed to an embryonic stem cell- (ESC-) like state. In 2006, Yamanaka's group announced that adult skin cells could be directly reprogrammed to become pluripotent stem cells using a combination of only four genes. They initially started with a list of 24 known pluripotency-associated genes expressed in ES cells. Ultimately, they succeeded in reprogramming mouse adult fibroblasts to an embryonic-like state using a cocktail of just four transcription factors, including octamer 3/4 (Oct3/4), SRY box-containing gene 2 (Sox2), Krüppel-like factor 4 (Klf4), and c-Myc [4]. The final reprogrammed cells were termed induced pluripotent stem cells (iPSCs). Later, two different research groups were able to effectively generate iPSCs from human somatic cells using slightly different combinations of genes, including Oct3/4, Sox2, Nanog, and LIN28A (LIN28) [5, 6]. Other scientists have been successful in generating iPSCs from fibroblasts [7], leukocytes [8], neural stem cells [9], hepatocytes [10], keratinocytes [11], pancreas cells [12], and cord blood cells [13]. Subsequently, reprogramming technology was successfully used to derive pluripotent cells from various other species, including the rhesus monkey [14], rat [15], cow [16], dog [17], sheep [18], goat [19], pig [20], horse [21], and buffalo [22].

In recent years, several methods have been successfully established for the generation of iPSCs, including virally induced iPSCs [12, 23–25], and nonvirally derived iPSCs using episomal vectors [26], minicircle vectors [27, 28], small molecules [29–33], transposon systems [34–37], mRNAs [38–40], microRNAs [41–44], and reprogramming proteins [45, 46]. However, it is worth mentioning that there are several hurdles that need to be overcome in order to develop safe iPSC technology for clinical trials. Lentiviral or retroviral vectors have the ability to integrate their transgene into the host genome. These transcriptionally silent proviruses can be reactivated at any time leading to oncogenesis. Adenoviral or episomal vectors facilitate transient expression of reprogramming factors without genomic integration. However, the reprogramming efficiency using episomal vectors is low and not completely free from the pitfalls of chromosomal disruption [47–49].

Among the experimental methods studied, transgene-free iPSC generation using reprogramming transcription factors has its own advantages and disadvantages. Generation of iPSCs by direct delivery of reprogramming proteins is a safe method that can be used for clinical trials. However, the reprogramming proteins are highly unstable and the reprogramming efficiency is low. Consequently, treatment with protein factors has to be performed repeatedly. This might lead to differences in reproducibility and is not an economical method. Thus, in this review we have attempted to compile available data in an effort to understand the importance of the ubiquitination and deubiquitination processes, which take place in core stem cell transcription factors and their application in developing an efficient method for cellular reprogramming.

## 3. Ubiquitin Proteasome Pathway

Ubiquitination is a process through which ubiquitin molecules are attached to protein substrates for protein degradation. It is one of the most important posttranslational modifications (PTMs) regulating the stability and functional activity of proteins. The ubiquitination process is orchestrated by a cascade of enzymes consisting of ubiquitin-activating enzymes (E1), ubiquitin-conjugating enzymes (E2), and ubiquitin ligases (E3) that mediate transfer of ubiquitin molecules onto targeted protein substrates [50–52].

**Figure 1:**
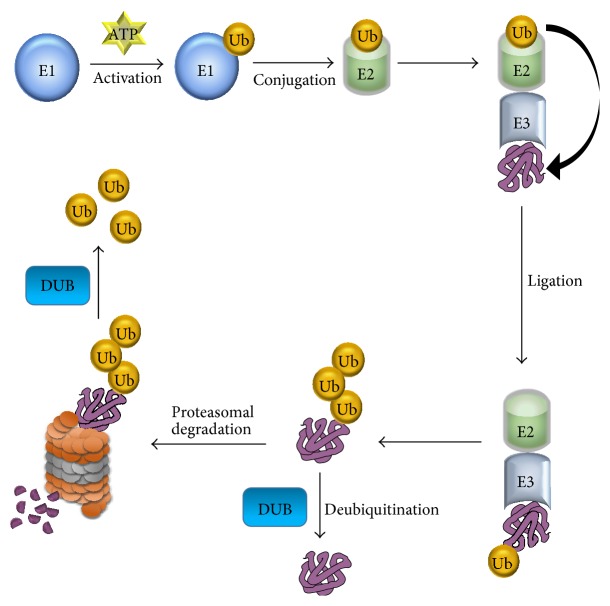
The ubiquitin proteasome system. The process of ubiquitination is catalyzed by an organized milieu of E1, E2, and E3 enzymes, which promote the ligation of a ubiquitin molecule to the lysine residues in the protein substrates. Lysine-48-linked polyubiquitination chain attached proteins are targeted to the 26S proteasome for protein degradation. DUB enzymes are involved in reversing ubiquitin conjugation and in the recycling of ubiquitin molecules through the ubiquitin proteasome pathway.

Ubiquitin conjugation initiates with the activation of a ubiquitin molecule by the E1 enzyme. During this process, an ATP-dependent thiol ester bond is formed between the C-terminus of the ubiquitin molecule and the active cysteine site of the E1 enzyme. Subsequently, ubiquitin is transferred to the E2 enzyme through a thioester linked E2-ubiquitin intermediate. Next, the E3 enzyme identifies and recruits the targeted substrate protein, interacts with the E2-ubiquitin intermediate, and catalyzes the transfer of ubiquitin to a lysine residue on the targeted protein. Finally, polyubiquitinated protein substrates are subjected to ATP-dependent hydrolysis by the 26S proteasome [50] (Figure 1).

Ubiquitin is a small molecule that attaches to protein substrates as a monomer or as polymers. Ubiquitin contains seven lysine residues within its sequence, lysine-6, lysine-11, lysine-27, lysine-29, lysine-33, lysine-48, and lysine-63, each of which can be utilized for the formation of ubiquitin-ubiquitin linkages called polyubiquitin chains [53]. Monoubiquitination occurs when a single ubiquitin molecule is attached to one lysine residue within the substrate, while polyubiquitination is the process through which a chain of ubiquitin molecules is attached to a specific lysine residue within the substrate. Usually, monoubiquitination of a protein serves as a signal for DNA repair, vesicle sorting, signal transduction, and receptor endocytosis [54–57], whereas polyubiquitination is mainly restricted to protein degradation and signal transduction [58].

**Figure 2:**
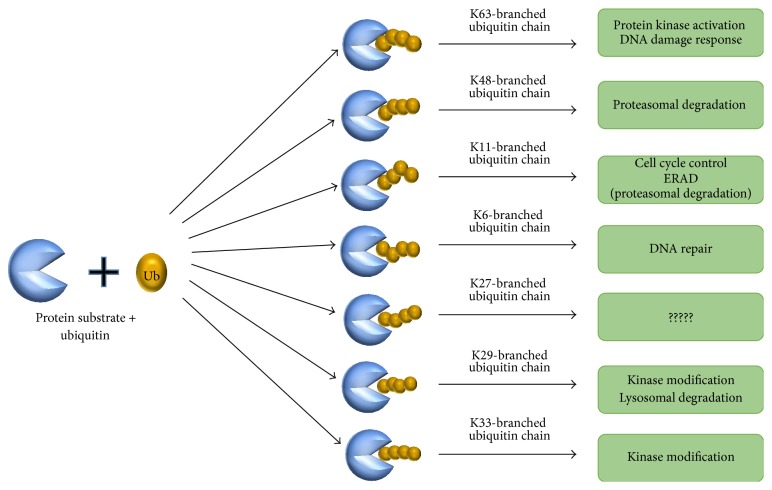
Ubiquitin modifications and their cellular functions. The attachment of ubiquitin molecules to one or more lysine residues results in polyubiquitination. Several types of polyubiquitin chains linked via lysine residues on the protein substrate are implicated in diverse cellular functions.

Ubiquitin chains are arranged in several different ways, which lead to distinct outcomes for the specific substrate. For instance, monoubiquitination and lysine-63 polyubiquitination have been linked to regulating protein activation or signal transduction. Lysine-6 and lysine-48 polyubiquitination target proteins for proteasomal degradation [59]. Several types of polyubiquitination and their cellular functions are illustrated in Figure 2. However, the ubiquitination process regulates several biological processes, such as cell cycle control, oncogenesis, immune response, transcriptional regulation, embryonic development, apoptosis, preimplantation, and intracellular signaling pathways [50].

## 4. Deubiquitination

**Figure 3:**
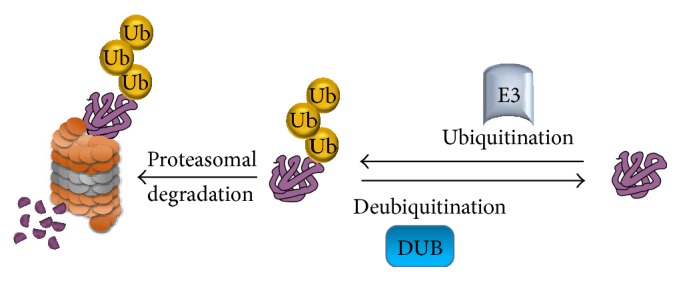
The ubiquitination and deubiquitination processes. A ubiquitin E3 ligase enzyme catalyzes the transfer of ubiquitin to lysine residue on the targeted protein and channels the protein to the 26S proteasome for protein degradation. Another class of enzyme, called deubiquitinating enzymes, that is able to reverse ubiquitin conjugation from protein substrates, thereby preventing proteolysis.

The process of cleaving ubiquitin molecules from ubiquitin-conjugated protein substrates by deubiquitinating enzymes (DUBs) is called deubiquitination. As every action provokes a reaction, all the major posttranslational modifications, including the ubiquitination process, can be reversed. Protein ubiquitination catalyzed by E3 ligases can be reversed by DUBs to prevent protein degradation. DUBs bind to the ubiquitin-based isopeptide bond, thus counteracting ubiquitin-protein ligase activity (Figure 3).

### 4.1. Deubiquitinating Enzymes and Their Classification

DUBs belong to a large family of proteases that reverse protein ubiquitination, which is an important process for maintaining cell homeostasis. DUBs can be divided into six families: (i) ubiquitin C-terminal hydrolases (UCH), (ii) ubiquitin specific processing proteases (USP), (iii) Jab1/Pab1/MPN domain-containing metalloenzymes (JAMM), (iv) Otu-domain ubiquitin aldehyde-binding proteins (OTU), (v) Ataxin-3/Josephin, and (vi) monocyte chemotactic protein-induced proteases (MCPIPs). Among these, USPs are the largest family, consisting of more than 50 members, each containing conserved domains and catalytic sites [50, 60–65]. The major cellular functions of DUBs are (i) processing of ubiquitin precursors, (ii) recycling ubiquitin molecules during ubiquitination, (iii) editing of ubiquitin chains, and (iv) reversal of ubiquitin conjugation [50, 66]. Thus, DUBs play a critical role in the regulation of the proteasomal pathway.

DUBs regulate a variety of cellular functions, such as the prevention of protein degradation, proteasome or lysosome dependent protein degradation, apoptosis, cell cycle progression, chromosome segregation, gene expression, DNA repair, kinase activation, and localization and degradation of signaling intermediates [50, 63–66]. However, DUB activity and specificity are determined by protein-protein interactions between protein complexes associated with DUBs, subcellular localization, alterations in their expression levels, and their differential activities in the various phases of the cell cycle [50, 66].

## 5. Reprogramming Somatic Cells to Pluripotency Using Core Transcriptional Factors

Pluripotent ESCs have the capacity to differentiate into several distinct cell lineages present in adult mammals. The status of pluripotency in ESCs is regulated by a few stem cell transcription factors. Among these transcriptional factors, a combination of core transcriptional factors, including Oct3/4, Sox2, c-Myc, Klf4, Nanog, and LIN28, has been proven to reprogram somatic cells into induced pluripotent stem cells [4, 5, 23, 67].

The POU transcription factor Oct3/4 has been found to be the most important Yamanaka factor in ES cell pluripotency and in the generation of iPSCs [68–70]. Oct3/4 expression appears in the early four- to eight-cell stage and declines as cells start to undergo differentiation into multiple lineages. The expression level of Oct3/4 plays a critical role in maintaining the pluripotent state of ES cells. A twofold higher than normal level of Oct3/4 expression induces ES differentiation into both mesoderm and endoderm [71] and a 1.5-fold higher than normal level in germ cells results in gonadal tumors [72]. Elevated levels of Oct3/4 alone can directly reprogram CD34+ cells into mesoderm progenitor cells or mesenchymal stem cells [73].

Sox2 is a member of a large protein family, characterized by their structure and sequence homology to the Sry (sex-determining region of chromosome Y) protein. Sox2 is considered to be a master regulator of both iPSCs and neural stem cells (NSCs). Sox2 has the potential to reestablish pluripotency in somatic cells by reprogramming them to iPSCs [4]. Increasing Sox2 levels in ESCs induces differentiation into the neural lineage [74, 75]. Interestingly, a recent report demonstrated that Sox2 alone is sufficient to directly reprogram fibroblasts into multipotent NSCs [76].

Klf4 is one of the Yamanaka transcription factors, sufficient to generate pluripotent stem cells from normal fibroblast cells [4]. However, although Klf4 alone is insufficient to induce reprogramming, it is essential for the generation of high quality iPSCs. Indeed, use of Klf4 results in the ability to generate iPSCs that are able to form high-contribution chimeras or efficiently generate “all-iPSC mice” by tetraploid (4n) complementation [77]. Klf4 acts as an upstream regulator of a large feed-forward loop that includes Oct3/4, Sox2, c-Myc, and Nanog, indicating the existence of a transcriptional hierarchy within the four reprogramming factors with both autoregulatory and feed-forward regulation. In addition, Klf4 enhances the core transcriptional network of iPSCs or ESCs and is also involved in mediating higher-order chromatin structure for the maintenance and induction of pluripotency.

c-Myc is considered to be a reprogramming inducer involved in the direct activation of pluripotent marker genes and in the maintenance of pluripotency in mouse ES cells [4]. c-Myc has been reported to be a universal amplifier of existing gene expression in lymphocytes, ESCs, and tumor cells through its accumulation on the promoter regions of active genes and also causes transcriptional amplification [78, 79]. However, c-Myc is avoided during reprogramming of cells due to its oncogenic behavior, which may lead to reactivation of Myc in progeny iPSCs, causing tumor formation [68, 80].

Nanog was initially reported to be the ENK gene (early embryo-specific NK) whose expression was specific to ES cells [81]. Later it was renamed Nanog by two independent groups [82, 83]. The expression of Nanog is confined to the inner cell mass of human blastocysts [84]. It is expressed at high levels in embryonic carcinoma cells and undifferentiated ESCs, and its expression level decreases upon ESC differentiation [82, 85]. A loss of pluripotency was reported in Nanog-deficient ESCs [86, 87], suggesting the importance of Nanog in maintenance of ES cell pluripotency.

Nanog was found to enhance reprogramming kinetics when it was included along with the Yamanaka factors during reprogramming of cells [67]. Nanog can enhance fusion-based reprogramming and also mouse epiblast stem cell reprogramming [88, 89]. Nanog is not required for the early stages of iPSC generation but is necessary for the final transition from the pre-iPSC state to the fully induced ground state [89]. Additionally, Esrrb, a direct downstream target of Nanog, has been found to be involved in the transition of pre-iPSCs to the pluripotent ground state [90].

LIN28 is also a reprogramming factor that, along with Oct3/4, Sox2, and Nanog, is able to successfully reprogram human somatic fibroblasts into iPSCs [5, 67]. Sox2, a pluripotency factor that directly binds to Lin28a, has been found to be critical in regulating Lin28a expression in single-cell gene expression during iPSCs reprogramming [91, 92]. Overexpression of Lin28a can reprogram adult hematopoietic stem and progenitor cells (HSPCs) into a fetal-like HSPCs, while Lin28b overexpression can expand neural crest progenitors, indicating its role in promoting stem cell self-renewal [93, 94].

## 6. Ubiquitination of Core Reprogramming Transcriptional Factors

**Figure 4:**
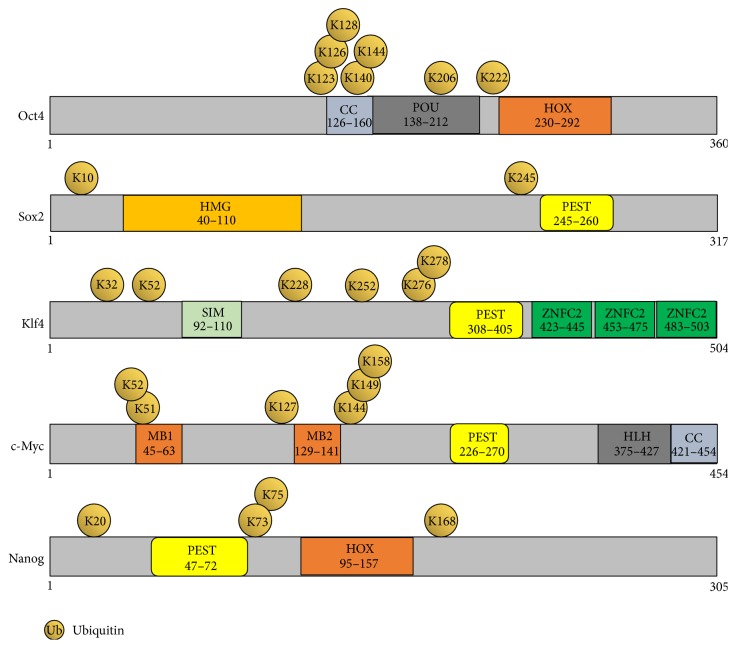
Schematic representation of the human Oct3/4, Sox2, Klf4, c-Myc, and Nanog proteins. Shown are the locations of both predicted and reported lysine sites for ubiquitination (lysine sites are predicted using the bioinformatics tool http://www.ubpred.org/).

Stem cell transcription factors are highly posttranscriptionally regulated at the levels of mRNA stability, translation, and protein stability. Among several different types of posttranslational modifications (PTMs), ubiquitination has emerged as a major regulator of protein turnover for these core stem cell transcription factors. Here, we have mapped the lysine sites present in core stem cell transcription factors that are predicted to undergo ubiquitination (Figure 4).

### 6.1. Ubiquitination of Oct3/4

Protein turnover of Oct3/4 is regulated by the ubiquitination process through direct binding of Wwp2, an E3 ubiquitin ligase, in mouse cells. In human ES cells, WWP2 targets Oct3/4 for protein degradation [95]. Protein degradation of Oct3/4 is rapid, with a relatively short half-life of about 90 minutes [96]. Wwp2 promotes both Oct3/4 ubiquitination and degradation to negatively regulate Oct3/4 transcriptional activity in ES cells [95]. Oct3/4 was also reported to undergo Lys-63 linked polyubiquitination and is targeted for protein degradation through the 26S proteasomal degradation pathway, which is catalyzed by the mouse E3 ligase Wwp2 [97]. Generally, poly-Ub chains linked by Lys-63 are not responsible for protein degradation signaling [98]. However, Wwp2 catalyzes Lys-63 polyubiquitination of Oct3/4 and these ubiquitinated Oct3/4 proteins have been tracked to the 26S proteasome for degradation [97]. The action of the E3 ligase Wwp2 on Oct3/4 ubiquitination was well detected during differentiation of ES cells. Upon treatment of cells with retinoid acid (RA), Oct3/4 ubiquitination was enhanced by Wwp2, indicating the role of Wwp2 in controlling Oct3/4 protein levels during the RA-induced differentiation process. The action of Wwp2 on Oct3/4 ubiquitination and degradation is dose-dependent; at high doses, the enzymatic activity of the E3 ligase is suppressed, due to its own ubiquitination, which occurs through an intramolecular mechanism. Thus, Wwp2 can control its own Ub ligase activity by undergoing homodimerization at higher concentrations and even signals Oct3/4 ubiquitination during the differentiation of ES cells [97].

Early studies showed that reprogramming efficiency is dependent on the continued expression of core stem cell transcription factors [99]. Recently, evidence has been growing that the efficiency of reprogramming is significantly influenced by the expression level of core stem cell transcription factors [99, 100]. The initiation phase of reprogramming has been observed in a majority of cells [99]. Interestingly, most cells then become refractory to reprogramming, with very few cells eventually progressing to the next phase of reprogramming. One of the possible reasons for this could be “innate immunity,” which signals for protein degradation. Thus, not all cells can be reprogrammed. This phenomenon can be overcome by additional overexpression of core stem cell transcription factors [99, 100]. Thus ubiquitination and the protein expression levels of core transcription factors play a significant role in cellular reprogramming.

Buckley et al. mapped the ubiquitinated protein landscape during ESC differentiation and induced pluripotency using a shotgun proteomics approach [100]. Additionally, using a ubiquitin-proteasome system-targeted RNAi screening method, they identified several regulators involved in the protein degradation of core stem cell transcription factors. Among these proteins, Psmd14, Ubr5, and Ddb1 played roles in regulating ESC self-renewal and pluripotency. Silencing of these three genes resulted in a significant reduction in the expression level of the pluripotency marker gene Oct3/4, coupled with morphological abnormalities in ESCs. Taken together, ubiquitination and the protein expression level of Oct3/4 play critical roles in maintaining self-renewal and reprogramming efficiency.

### 6.2. Ubiquitination of Klf4

Ubiquitination of Klf4 is an important posttranslational modification and is responsible for regulating its protein turnover in the cells. Chen et al. reported that serum stimulation downregulates Klf4 protein level [101]. Variations in Klf4 protein levels during serum stimulation were found to be associated with proteasomal function and were confirmed through the use of proteasomal inhibitor MG132. MG132-pretreated cells failed to show a decrease in Klf4 protein levels upon serum stimulation. Klf4 undergoes rapid protein degradation and has a relatively short half-life of about 120 minutes. MG132-treated cells were partially refractory to Klf4 protein degradation, resulting in an extension of its half-life. Ubiquitinated Klf4 conjugates were observed at a high level in proliferating cells as compared with serum-starved cells, suggesting the importance of ubiquitination in serum-mediated degradation [101].

Recently, Hu and Wan showed that Klf4 expression was downregulated in response to TGF-*β*-signaling, which was mediated by the ubiquitin-proteasomal pathway (UPP) [102]. The half-life of Klf4 was significantly reduced, suggesting that TGF-*β* enhances Klf4 protein turnover; this finding was confirmed through the inhibitory effect of MG132 on TGF-*β*-induced Klf4 protein degradation. Furthermore, Cdh1/APC, a putative E3 ubiquitin ligase, was found to interact with Klf4 and to regulate TGF-*β*-induced Klf4 proteolysis. Mutation of the two destruction boxes within Klf4 resulted in reduced ubiquitination and subsequently resulted in protein stabilization. Thus, stabilized Klf4 impaired TGF-*β*-induced transcriptional activation and further antagonized TGF-*β*-induced growth inhibition [102]. Phosphorylation of Klf4 was reported to enhance the ubiquitination and protein degradation of Klf4 [103]. Klf4 phosphorylation by ERK1 recruits *β*TrCP1 or *β*TrCP2, an F-box protein with E3 ubiquitin ligase activity, to its N-terminal region and signals for protein degradation of Klf4 [103]. Thus, treatment with inhibitors of E3 ligases, such as Cdh1/APC or *β*TrCP1, which are known to interact with Klf4 and trigger protein degradation of Klf4, might enhance self-renewal capacity and enable Klf4 to reprogram embryonic fibroblasts more efficiently.

### 6.3. Ubiquitination of c-Myc

Ubiquitination and proteolysis of c-Myc are also important posttranslational modifications regulating the stability and function of c-Myc. Unlike other defined transcription factors, c-Myc is an unstable protein exhibiting a half-life of about 20–30 minutes [104]. UPP is responsible for the degradation of many short-lived regulatory proteins* in vivo* [105]. Inhibition of proteasome activity using different proteasome inhibitors was found to enhance the stability of the c-Myc protein [106–109]. In this context, several research groups have examined the domains or regions responsible for c-Myc ubiquitination and protein degradation.

Flinn et al. reported that the regions between amino acids 45 and 63 of Myc Box I (MBI) and amino acids 126 to 144 of MBII are degrons responsible for c-Myc proteolysis in both yeast and mammalian cells [110]. Salghetti et al. conducted ubiquitination assays on deletion constructs of c-Myc and concluded that 128 amino acids in the N-terminal region contain the Myc degron signal for proteolysis [107]. Indeed, 94 amino acids from the N-terminal region of c-Myc contribute to destabilization and 147 amino acids from the N-terminal region of c-Myc result in ubiquitination [107]. In contrast to previous results, Gregory and Hann identified the primary degrons as consisting of amino acids of 127–158 of c-Myc and also found that the N-terminal 100 amino acids are also responsible for c-Myc stabilization, leading to the prediction that the secondary degron might be located within the N-terminal 100 amino acids [108]. A deletion construct of c-Myc lacking MBII (c-MycSΔ 106-143) in which the primary degron lies was also efficiently degraded, suggesting that the primary degron extends beyond MBII [108]. Thus, MBI and MBII may serve as binding regions for several ubiquitin ligases that regulate proteolysis, rather than directly signaling protein degradation. In addition, a PEST sequence between amino acids 226 and 270 was shown to be responsible for rapid c-Myc degradation but did not have any effect on c-Myc ubiquitination [108]. PEST motif sequences are enriched in proline (P), glutamic acid (E), serine (S), threonine (T), and aspartic acid (D) residues and have been implicated as degradation signals [111, 112]. Several reports have shown the importance of PEST motifs in tracking proteins required for the ubiquitin-mediated proteasomal pathway [112]. It has also been reported that the PEST region is involved in signaling calpain-mediated proteolysis [113]. Calpain activation induces rapid cleavage of c-Myc* in vivo* and* in vitro* [114], but the relationship between the PEST motif of c-Myc and calpain-mediated c-Myc proteolysis has not yet been determined.

S-phase kinase associated protein (Skp) 2, an F-box protein in the ubiquitin ligase complex, was the first ubiquitin ligase for c-Myc identified in yeast cells [115, 116]. Skp2 was found to interact with multiple regions of c-Myc, mainly between amino acids 129–147 (MBII) and amino acids 379–418 [115, 116]. Skp2 significantly enhances the ubiquitination status of c-Myc, particularly in the region between amino acids 129–147, which contains one lysine residue (K144) [115]. Additionally, Skp2-mediated ubiquitination of c-Myc has been shown to regulate c-Myc transcriptional activity. Skp2 was shown to induce a threefold increase in c-Myc transcriptional activity in Gal4 transactivation assays [115]. Skp2 was also found to induce the *α*-prothymosin promoter and cause a synergistic effect [116]. In turn, Skp2-induced activation of several promoters requires c-Myc. The interaction between Skp2 and c-Myc leads to diminished c-Myc protein levels due to increased c-Myc turnover. In particular, Skp2-mediated c-Myc turnover was observed at the G1 to S phase transition during the activation of resting lymphocytes. Apart from the Skp2 promotion of c-Myc degradation, Skp2 is also involved in regulating c-Myc's cellular function by enhancing c-Myc-induced S phase entry [116].

Fbw7, a component of the SCF ubiquitin ligase complex containing the F-box substrate recognizing protein, was also shown to promote c-Myc turnover* in vivo* and c-Myc ubiquitination* in vitro* [117]. All three isoforms of the Fbw7 gene (Fbw7*α*, Fbw7*β*, and Fbw7*γ*) can negatively regulate c-Myc turnover. In particular, addition of the proteasomal inhibitor MG132 reversed c-Myc turnover, suggesting that c-Myc turnover is proteasome-dependent. Unlike Skp2, Fbw7 decreased c-Myc transcriptional activity in a dose-dependent manner in c-Myc transactivation assays [117]. Fbw7 binds with both Thr-58 and Thr-58/Ser-62 doubly phosphorylated c-Myc peptides and the interaction with Fbw7 that mediates c-Myc protein turnover is dependent on Thr-58 phosphorylation of c-Myc. In addition, inhibition of GSK3 prevents Fbw7-mediated c-Myc proteolysis, indicating that Fbw7-driven c-Myc turnover depends on phosphorylation of c-Myc on Thr-58 by GSK-3. Thus, Thr-58 phosphorylation-dependent Fbw7 ubiquitination of c-Myc is not required for c-Myc transcriptional activity, as was reported for Skp2; instead it might be involved in c-Myc-mediated apoptosis. Another study, showed that the region spanning amino acids 127–189 contains a JNK binding domain and also found that JNK interacts with c-Myc and promotes c-Myc ubiquitination and degradation* in vivo* and* in vitro* [118]. Thus, JNK might associate with other ubiquitin ligases to enhance the ubiquitination and degradation of c-Myc.

### 6.4. Ubiquitination of Sox2

Recent studies involving large-scale analyses of phosphorylation in human ES cells have revealed that Sox2 proteins are phosphorylated. Four potential phosphorylation sites have been mapped within the Sox2 protein at Ser-246, Ser-249, Ser-250, and Ser-251 [119, 120]. Stabilization of the Sox2 protein upon Sox2 phosphorylation is in turn regulated by suppression of ubiquitin-mediated protein degradation. Phosphorylation of Sox2 at Thr-118 enhances protein stability by antagonizing Sox2 protein degradation; this is not seen when mutant T118A Sox2 is utilized. Phosphorylation of Sox2 not only promotes Sox2 stability by preventing protein degradation but also enhances the self-renewal capacity of mouse ESCs, which enables Sox2 to reprogram mouse embryonic fibroblasts more efficiently [121].

### 6.5. Ubiquitination of Nanog

Regulation of human Nanog by UPP was demonstrated by treatment with MG132, a proteasomal inhibitor, which resulted in increased endogenous ubiquitination of Nanog [122]. In addition, Nanog showed conjugation with both Lys48- and Lys63-branched polyubiquitin chains* in vivo*. Nanog, which has a PEST motif sequence from amino acids 47 to 72 at its N-terminal region, was shown to target proteins for ubiquitination. However, a PEST motif-deleted Nanog protein was found to be more stable due to suppression of the ubiquitination process. Thus, the PEST motif sequence appears to be the signaling factor in Nanog protein degradation. The level of endogenous Nanog in human ES cells can be increased by inhibiting proteasome activity and also by regulating its half-life. Nanog was reported to have a relatively short half-life of about 120 minutes in human ESCs. Pretreatment of human ESCs with the proteasome inhibitor MG132 increased Nanog protein stability and extended its half-life [122]. Thus, fluctuations in the expression of Nanog in mouse ESCs [87] may be due to regulation of its protein degradation during pluripotency. It has been reported that Nanog can be phosphorylated at four Ser/Thr-Pro motifs, which facilitates the interaction between Nanog and prolyl isomerase Pin1. As a functional consequence of the interaction between Nanog and Pin1, Nanog protein degradation is suppressed, resulting in stabilization of the Nanog protein [123]. Thus, increasing Pin1 activity enhances the capability of Nanog to maintain self-renewal and enables Nanog to reprogram mouse embryonic fibroblasts more efficiently.

### 6.6. Critical Lysine Residues for Protein Stabilization of Core Stem Cell Transcription Factors

Based on a bioinformatics analysis, core stem cell transcription factors contain several predicted ubiquitination sites at lysine residues, as summarized in Figure 4. Replacing the predicted sites of ubiquitination, which are responsible for protein degradation and transactivation suppression, in the core stem cell transcription factors might improve the stability of these proteins when used for protein-induced iPSC generation. Despite some drawbacks, such as difficulties associated with the purification of stable proteins and low efficiency, the protein-induced iPSC generation method is very promising for the production of patient-specific iPSCs. Thus, expression and purification of modified versions of core transcription factor proteins with longer half-lives might improve protein-induced iPSC generation efficiency.

Using this approach, we predicted the lysine sites on the Klf4 protein potentially responsible for protein degradation by referring to two bioinformatics databases, UbPred (http://www.ubpred.org/) and NetChop (http://www.cbs.dtu.dk/services/NetChop/). Several Klf4 deletion fragments were analyzed for ubiquitination and the critical lysine residues that signal protein degradation were replaced with arginine residues. Finally, we identified the critical lysine site at position 232 as being responsible for the high level of ubiquitination. A lysine 232 mutant of Klf4 protein had a longer half-life and increased protein stability [124]. This mutant Klf4 protein is being investigated as a way to improve reprogramming efficiency.

Given that c-Myc is an unstable protein with a half-life of about 20–30 minutes [104], it is essential to identify the lysine residue on c-Myc that is responsible for protein degradation. Taken together, the ability to establish stable core transcription factors without affecting their reprogramming efficiency and the use of these proteins for protein-induced iPSC generation might be a great contribution to the field of cellular reprogramming.

## 7. Deubiquitinating Enzymes Regulating Core Stem Cell Transcription Factors

The role of posttranslational regulation in stem cell maintenance and cellular reprogramming has been extensively studied. A key mechanism of posttranslational modification is ubiquitination by the UPS, which regulates protein turnover of core stem cell transcription factors. Although several physiological functions of the UPS in ESC pluripotency and cellular reprogramming have been reported, there is limited information on the functions of DUBs in stem cell maintenance and cellular reprogramming. However, recent studies on a few DUB candidates, mainly USP22, Psmd14, and USP44, have revealed the role of DUBs in regulating stem cell transcription factors and their influence of the efficiency of cellular reprogramming.

### 7.1. USP22

USP22 is a cysteine protease that acts as a transcriptional activator or repressor. USP22 was found to hydrolyze monoubiquitin tagged to uH2A and to antagonize PcG or hydrolyze monoubiquitin from uH2B to regulate MLL-trithorax-mediated trimethylation of histone H3 lysine-4 [125–129]. Recently, several lines of evidence have proven that USP22 plays a major role in stem cell function.* USP22* can be considered a cancer stem cell marker gene due to its activity, which facilitates aggressive cellular phenotypes, including metastatic potential and resistance to therapy [130, 131]. The USP22 locus has been found to be actively transcribed in both human ESCs and iPSCs. Additionally, the histone H3 lysine-4 trimethyl epigenetic marker is recruited to the USP22 promoter, which is also occupied by the core pluripotency factor KLF4, suggesting its role in stem cell pluripotency and differentiation [132]. USP22 has also been shown to be essential for embryonic development in mice [131].

Recent evidence has revealed that USP22 regulates core pluripotency factors, including c-Myc and Sox2. USP22 was identified as an essential cofactor for the stem cell transcription factor Myc in the regulation of transcription of Myc target genes [128]. Sussman et al. showed that USP22 is induced as the differentiation process of ESCs progresses. The expression level of USP22 is critical during ESC differentiation; its ectopic expression can trigger differentiation even in the absence of other differentiation signals. Depletion of USP22 resulted in defects in the transcription of genes related to all three germ layers, indicating its requirement for proper ESC differentiation into all three germ layers [133]. During ESC differentiation, USP22 acts as a transcriptional repressor of the* Sox2* locus. USP22 has been found to be located directly on the* Sox2* promoter and catalyzes deubiquitination of H2B and attenuates* Sox2* transcription. By contrast, depletion of RNF20, the E3 ligase of H2B, opposes the effect of USP22 on* Sox2* transcription [133]. Thus, USP22 plays a pivotal role in the efficient differentiation of ESCs by repressing Sox2 and allowing ESCs to transition from a state of self-renewal to lineage-specific differentiation pathways.

### 7.2. Psmd14

Psmd14 was initially identified as a component of the 19S proteasome lid [134]. Psmd14 is highly expressed in pluripotent ESCs, whereas its expression level decreases significantly upon differentiation. Psmd14 was one of the DUB candidates identified along with USP9X when UPS-targeted siRNA screening was performed to identify genes required to maintain ES cell self-renewal and pluripotency [100]. Depletion of Psmd14 leads to a significant decrease in Oct4 protein expression coupled with abnormal ESCs morphology. Psmd14 was found to interact with the majority of the 19S proteasome lid, including Psmd3, Psmd6, Psmd7, Psmd11, Psmd12, and Psmd13, in ESCs. Depletion of Psmd14 did not alter the overall stoichiometry of the 26S proteasome as no significant changes were observed in the expression of its interacting partners or in the components of the proteasome lid. However, there was a defect in proteasome activity leading to accumulation of both K48- and K63-linked polyubiquitinated proteins. In addition, there was a loss of Oct3/4 expression and morphological changes consistent with ES differentiation. Finally, Psmd14 expression was found to be absolutely essential for generating iPSCs. When Oct3/4, Klf4, Sox2, and c-Myc expressing MEFs were transduced with virus expressing shRNAs against Psmd14, the MEFs expressing Psmd14 shRNAs failed to reprogram and generate iPSCs [100]. Thus, Psmd14 is strongly considered to be an important candidate required for iPSC generation.

### 7.3. Other DUBs

Based on genome-scale location analysis, several DUBs have been reported to play roles in transcriptional regulation of human embryonic stem cells. These DUBs bind to the promoter regions of core embryonic transcription factors, such as Oct4, Sox2, and Nanog [135]. USP44 and USP7 were found to bind to the Oct4 promoter. USP25, USP44, USP49, and USP7 bind to the Sox2 promoter, while USP10, USP16, USP3, USP37, USP44, and USP7 bind to the Nanog promoter. In addition, USP9X was also found in mouse and human stem cells, including embryonic and neural stem cells or neuronal progenitors, hematopoietic stem cells, and adult epidermal stem cells [136, 137]. However, the mechanism of Oct4, Sox2, and Nanog protein regulation by DUBs and their role in stem cell differentiation and cellular reprogramming remains unidentified.

Recently, the functions of USP7 and USP44 were linked to stem cell maintenance and differentiation [138, 139]. REST is a stem cell transcription factor and its protein level is critical during neural differentiation. USP7 interacts and stabilizes the REST protein by blocking SCF*β*-TrCP-mediated ubiquitination, thereby promoting the maintenance of stemness [138]. USP44 acts as a negative regulator of H2B ubiquitination during stem cell differentiation. Depletion of USP44 results in an increase in H2B ubiquitination, whereas monoubiquitination of H2B is known to increase during stem cell differentiation, suggesting that an optimum expression level of USP44 is required for ESC differentiation [139].

## 8. Conclusions

A growing body of evidence has proven that core stem cell transcription factors regulating ESC self-renewal and stem cell maintenance, such as Oct3/4, c-Myc, Sox2, Klf4, and Nanog, are ubiquitinated by several different E3 ubiquitin ligases. Indeed, E3 ligases have been shown to have a negative influence during the generation of iPSCs by mediating protein degradation of core transcription factors. Based on recent reports, it is likely that each stem cell transcription factor can be deubiquitinated by specific DUBs. Thus, stem cell transcription factors are regulated by both ubiquitination and deubiquitination at the posttranslational level. Therefore, identification of the DUBs that reverse the proteolysis of stem cell transcription factors will be important to our understanding of the molecular mechanisms of cell fate determination of ESCs.

**Figure 5:**
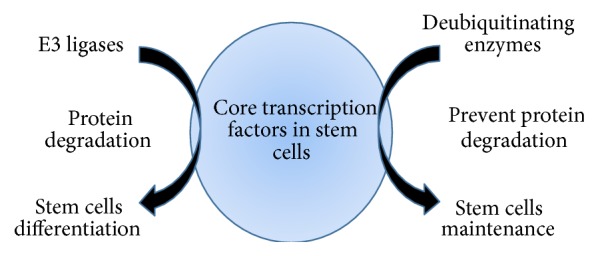
Schematic representation of the roles of E3 ligases and deubiquitinating enzymes in regulating stem cell differentiation and stem cell maintenance. Ubiquitination of core stem cell transcription factors by E3 ligases mediates stem cell differentiation. Deubiquitination of the core stem cell transcription factors by DUBs mediates stem cell maintenance.

Balanced control over the ubiquitination and deubiquitination processes of stem cell transcription factors determines the fate of the stem cells with respect to differentiation or the maintenance of pluripotency. Ubiquitination of stem cell transcription factors by E3 ligases results in stem cell differentiation, while activation of DUBs prevents proteolysis by stabilizing stem cell transcription factors and promoting stem cell maintenance (Figure 5). As suggested by the available data, methods to control the action of E3 ubiquitin ligases on stem cell transcription factors during cellular reprogramming might improve the reprogramming efficiency. One possible way of blocking the interaction between E3 ubiquitin ligases and stem cell transcription factors would be to use protein inhibitors that specifically target the E3 ubiquitin ligases for the stem cell transcription factors utilized during the cellular reprogramming process. Alternatively, we can screen for potential DUBs that regulate the protein levels of core stem cell transcription factors. Delivering a combination of potential DUBs that regulate protein turnover of stem cell transcription factors along with the core transcription factors during cellular reprogramming might enhance the efficiency of cellular reprogramming. Taken together, utilizing this paradigm of reciprocal posttranslational control by DUBs in stem cell regulatory networks during iPSC generation might significantly improve cellular reprogramming efficiency, thereby leading to an advanced and novel route for cellular reprogramming.

## Abbreviations

PTM: Posttranslational modification
DUB: Deubiquitinating enzyme
ICM: Inner cell mass
ESCs: Embryonic stem cells
hESCs: Human embryonic stem cells
iPSCs: Induced pluripotent stem cells
NSCs: Neural stem cells
HSPCs: Hematopoietic stem and progenitor cells
RA: Retinoid acid
UPP: Ubiquitin-proteasomal pathway
UCH: Ubiquitin C-terminal hydrolases
USP: Ubiquitin specific processing proteases
JAMM: Jab1/Pab1/MPN domain-containing metalloenzymes
OUT: Otu-domain ubiquitin aldehyde-binding proteins.

## References

[1] Thomson J. A., Itskovitz-Eldor J., Shapiro S. S. (1998). Embryonic stem cell lines derived from human blastocysts. *Science*.

[2] Cowan C. A., Klimanskaya I., McMahon J. (2004). Derivation of embryonic stem-cell lines from human blastocysts. *The New England Journal of Medicine*.

[3] Lensch M. W., Daley G. Q. (2004). Origins of mammalian hematopoiesis: in vivo paradigms and in vitro models. *Current Topics in Developmental Biology*.

[4] Takahashi K., Yamanaka S. (2006). Induction of pluripotent stem cells from mouse embryonic and adult fibroblast cultures by defined factors. *Cell*.

[5] Yu J., Vodyanik M. A., Smuga-Otto K. (2007). Induced pluripotent stem cell lines derived from human somatic cells. *Science*.

[6] Park I.-H., Zhao R., West J. A. (2008). Reprogramming of human somatic cells to pluripotency with defined factors. *Nature*.

[7] Lowry W. E., Richter L., Yachechko R. (2008). Generation of human induced pluripotent stem cells from dermal fibroblasts. *Proceedings of the National Academy of Sciences of the United States of America*.

[8] Hanna J., Markoulaki S., Schorderet P. (2008). Direct reprogramming of terminally differentiated mature B lymphocytes to pluripotency. *Cell*.

[9] Kim J. B., Greber B., Arazo-Bravo M. J. (2009). Direct reprogramming of human neural stem cells by OCT4. *Nature*.

[10] Aoi T., Yae K., Nakagawa M. (2008). Generation of pluripotent stem cells from adult mouse liver and stomach cells. *Science*.

[11] Aasen T., Raya A., Barrero M. J. (2008). Efficient and rapid generation of induced pluripotent stem cells from human keratinocytes. *Nature Biotechnology*.

[12] Stadtfeld M., Brennand K., Hochedlinger K. (2008). Reprogramming of pancreatic beta cells into induced pluripotent stem cells. *Current Biology*.

[13] Takenaka C., Nishishita N., Takada N., Jakt L. M., Kawamata S. (2010). Effective generation of iPS cells from CD34^+^ cord blood cells by inhibition of p53. *Experimental Hematology*.

[14] Liu H., Zhu F., Yong J. (2008). Generation of induced pluripotent stem cells from adult rhesus monkey fibroblasts. *Cell Stem Cell*.

[15] Liao J., Cui C., Chen S. (2009). Generation of induced pluripotent stem cell lines from adult rat cells. *Cell Stem Cell*.

[16] Han X., Han J., Ding F. (2011). Generation of induced pluripotent stem cells from bovine embryonic fibroblast cells. *Cell Research*.

[17] Shimada H., Nakada A., Hashimoto Y., Shigeno K., Shionoya Y., Nakamura T. (2010). Generation of canine induced pluripotent stem cells by retroviral transduction and chemical inhibitors. *Molecular Reproduction and Development*.

[18] Bao L., He L., Chen J. (2011). Reprogramming of ovine adult fibroblasts to pluripotency via drug-inducible expression of defined factors. *Cell Research*.

[19] Ren J., Pak Y., He L. (2011). Generation of hircine-induced pluripotent stem cells by somatic cell reprogramming. *Cell Research*.

[20] Esteban M. A., Xu J., Yang J. (2009). Generation of induced pluripotent stem cell lines from Tibetan miniature pig. *The Journal of Biological Chemistry*.

[21] Nagy K., Sung H. K., Zhang P. (2011). Induced pluripotent stem cell lines derived from equine fibroblasts. *Stem Cell Reviews and Reports*.

[22] Deng Y., Liu Q., Luo C. (2012). Generation of induced pluripotent stem cells from buffalo (*Bubalus bubalis*) fetal fibroblasts with buffalo defined factors. *Stem Cells and Development*.

[23] Takahashi K., Tanabe K., Ohnuki M. (2007). Induction of pluripotent stem cells from adult human fibroblasts by defined factors. *Cell*.

[24] Kane N. M., Nowrouzi A., Mukherjee S. (2010). Lentivirus-mediated reprogramming of somatic cells in the absence of transgenic transcription factors. *Molecular Therapy*.

[25] Fusaki N., Ban H., Nishiyama A., Saeki K., Hasegawa M. (2009). Efficient induction of transgene-free human pluripotent stem cells using a vector based on Sendai virus, an RNA virus that does not integrate into the host genome. *Proceedings of the Japan Academy Series B, Physical and Biological Sciences*.

[26] Yu J., Hu K., Smuga-Otto K. (2009). Human induced pluripotent stem cells free of vector and transgene sequences. *Science*.

[27] Jia F., Wilson K. D., Sun N. (2010). A nonviral minicircle vector for deriving human iPS cells. *Nature Methods*.

[28] Yoshida Y., Takahashi K., Okita K., Ichisaka T., Yamanaka S. (2009). Hypoxia enhances the generation of induced pluripotent stem cells. *Cell Stem Cell*.

[29] Ichida J. K., Blanchard J., Lam K. (2009). A small-molecule inhibitor of tgf-*β* signaling replaces sox2 in reprogramming by inducing nanog. *Cell Stem Cell*.

[30] Jung D.-W., Kim W.-H., Williams D. R. (2014). Reprogram or reboot: small molecule approaches for the production of induced pluripotent stem cells and direct cell reprogramming. *ACS Chemical Biology*.

[31] Lee C. H., Kim J.-H., Lee H. J. (2011). The generation of iPS cells using non-viral magnetic nanoparticlebased transfection. *Biomaterials*.

[32] Shi Y., Do J. T., Desponts C., Hahm H. S., Schöler H. R., Ding S. (2008). A combined chemical and genetic approach for the generation of induced pluripotent stem cells. *Cell Stem Cell*.

[33] Desponts C., Ding S. (2010). Using small molecules to improve generation of induced pluripotent stem cells from somatic cells.. *Methods in Molecular Biology*.

[34] Grabundzija I., Wang J., Sebe A. (2013). Sleeping Beauty transposon-based system for cellular reprogramming and targeted gene insertion in induced pluripotent stem cells. *Nucleic Acids Research*.

[35] Kues W. A., Herrmann D., Barg-Kues B. (2013). Derivation and characterization of sleeping beauty transposon-mediated porcine induced pluripotent stem cells. *Stem Cells and Development*.

[36] Talluri T. R., Kumar D., Glage S. (2014). Non-viral reprogramming of fibroblasts into induced pluripotent stem cells by Sleeping Beauty and piggyBac transposons. *Biochemical and Biophysical Research Communications*.

[37] Woltjen K., Hämäläinen R., Kibschull M., Mileikovsky M., Nagy A. (2011). Transgene-free production of pluripotent stem cells using piggyBac transposons. *Methods in Molecular Biology*.

[38] Warren L., Manos P. D., Ahfeldt T. (2010). Highly efficient reprogramming to pluripotency and directed differentiation of human cells with synthetic modified mRNA. *Cell Stem Cell*.

[39] Plews J. R., Li J., Jones M. (2010). Activation of pluripotency genes in human fibroblast cells by a novel mRNA based approach. *PLoS ONE*.

[40] Yoshioka N., Gros E., Li H.-R. (2013). Efficient generation of human iPSCs by a synthetic self-replicative RNA. *Cell Stem Cell*.

[41] Anokye-Danso F., Trivedi C. M., Juhr D. (2011). Highly efficient miRNA-mediated reprogramming of mouse and human somatic cells to pluripotency. *Cell Stem Cell*.

[42] Card D. A. G., Hebbar P. B., Li L. (2008). Oct4/Sox2-regulated miR-302 targets cyclin D1 in human embryonic stem cell. *Molecular and Cellular Biology*.

[43] Lin S.-L., Chang D. C., Chang-Lin S. (2008). Mir-302 reprograms human skin cancer cells into a pluripotent ES-cell-like state. *RNA*.

[44] Miyoshi N., Ishii H., Nagano H. (2011). Reprogramming of mouse and human cells to pluripotency using mature microRNAs. *Cell Stem Cell*.

[45] Kim D., Kim C.-H., Moon J.-I. (2009). Generation of human induced pluripotent stem cells by direct delivery of reprogramming proteins. *Cell Stem Cell*.

[46] Zhou H., Wu S., Joo J. Y. (2009). Generation of induced pluripotent stem cells using recombinant proteins. *Cell Stem Cell*.

[47] Stadtfeld M., Nagaya M., Utikal J., Weir G., Hochedlinger K. (2008). Induced pluripotent stem cells generated without viral integration. *Science*.

[48] Zhou W., Freed C. R. (2009). Adenoviral gene delivery can reprogram human fibroblasts to induced pluripotent stem cells. *Stem Cells*.

[49] Yamanaka S. (2009). A fresh look at iPS cells. *Cell*.

[50] Amerik A. Y., Hochstrasser M. (2004). Mechanism and function of deubiquitinating enzymes. *Biochimica et Biophysica Acta*.

[51] Ciechanover A., Shkedy D., Oren M., Bercovich B. (1994). Degradation of the tumor suppressor protein p53 by the ubiquitin-mediated proteolytic system requires a novel species of ubiquitin-carrier protein, E2. *The Journal of Biological Chemistry*.

[52] Ciechanover A., Heller H., Elias S., Haas A. L., Hershko A. (1980). ATP-dependent conjugation of reticulocyte proteins with the polypeptide required for protein degradation. *Proceedings of the National Academy of Sciences of the United States of America*.

[53] Komander D. (2009). The emerging complexity of protein ubiquitination. *Biochemical Society Transactions*.

[54] Sigismund S., Polo S., Di Fiore P. P. (2004). Signaling through monoubiquitination. *Current Topics in Microbiology and Immunology*.

[55] Sun L., Chen Z. J. (2004). The novel functions of ubiquitination in signaling. *Current Opinion in Cell Biology*.

[56] Sadowski M., Suryadinata R., Tan A. R., Roesley S. N. A., Sarcevic B. (2012). Protein monoubiquitination and polyubiquitination generate structural diversity to control distinct biological processes. *IUBMB Life*.

[57] Ramanathan H. N., Ye Y. (2012). Cellular strategies for making monoubiquitin signals. *Critical Reviews in Biochemistry and Molecular Biology*.

[58] Komander D., Rape M. (2012). The ubiquitin code. *Annual Review of Biochemistry*.

[59] Ikeda F., Dikic I. (2008). Atypical ubiquitin chains: new molecular signals. ‘Protein Modifications: Beyond the Usual Suspects’ review series. *EMBO Reports*.

[60] Baek K.-H. (2006). Cytokine-regulated protein degradation by the ubiquitination system. *Current Protein and Peptide Science*.

[61] Baek K.-H., Kim M.-S., Kim Y.-S., Shin J.-M., Choi H.-K. (2004). DUB-1A, a novel subfamily member of deubiquitinating enzyme, is polyubiquitinated and cytokine inducible in B-lymphocytes. *Journal of Biological Chemistry*.

[62] Lim K.-H., Ramakrishna S., Baek K.-H. (2013). Molecular mechanisms and functions of cytokine-inducible deubiquitinating enzymes. *Cytokine and Growth Factor Reviews*.

[63] Ramakrishna S., Kim K.-S., Baek K.-H. (2014). Posttranslational modifications of defined embryonic reprogramming transcription factors. *Cellular Reprogramming*.

[64] Ramakrishna S., Suresh B., Baek K.-H. (2011). The role of deubiquitinating enzymes in apoptosis. *Cellular and Molecular Life Sciences*.

[65] Ramakrishna S., Suresh B., Baek K.-H. (2015). Biological functions of hyaluronan and cytokine-inducible deubiquitinating enzymes. *Biochimica et Biophysica Acta*.

[66] Reyes-Turcu F. E., Ventii K. H., Wilkinson K. D. (2009). Regulation and cellular roles of ubiquitin-specific deubiquitinating enzymes. *Annual Review of Biochemistry*.

[67] Hanna J., Saha K., Pando B. (2009). Direct cell reprogramming is a stochastic process amenable to acceleration. *Nature*.

[68] Okita K., Ichisaka T., Yamanaka S. (2007). Generation of germline-competent induced pluripotent stem cells. *Nature*.

[69] Rosner M. H., Vigano M. A., Ozato K. (1990). A POU-domain transcription factor in early stem cells and germ cells of the mammalian embryo. *Nature*.

[70] Okamoto K., Okazawa H., Okuda A., Sakai M., Muramatsu M., Hamada H. (1990). A novel octamer binding transcription factor is differentially expressed in mouse embryonic cells. *Cell*.

[71] Niwa H., Miyazaki J.-I., Smith A. G. (2000). Quantitative expression of Oct-3/4 defines differentiation, dedifferentiation or self-renewal of ES cells. *Nature Genetics*.

[72] Looijenga L. H. J., Stoop H., de Leeuw H. P. J. C. (2003). POU5F1 (OCT3/4) identifies cells with pluripotent potential in human germ cell tumors. *Cancer Research*.

[73] Meng X., Su R.-J., Baylink D. J. (2013). Rapid and efficient reprogramming of human fetal and adult blood CD34^+^ cells into mesenchymal stem cells with a single factor. *Cell Research*.

[74] Kopp J. L., Ormsbee B. D., Desler M., Rizzino A. (2008). Small increases in the level of Sox2 trigger the differentiation of mouse embryonic stem cells. *STEM CELLS*.

[75] Zhao S., Nichols J., Smith A. G., Li M. (2004). SoxB transcription factors specify neuroectodermal lineage choice in ES cells. *Molecular and Cellular Neuroscience*.

[76] Ring K. L., Tong L. M., Balestra M. E. (2012). Direct reprogramming of mouse and human fibroblasts into multipotent neural stem cells with a single factor. *Cell Stem Cell*.

[77] Guo G., Yang J., Nichols J. (2009). Klf4 reverts developmentally programmed restriction of ground state pluripotency. *Development*.

[78] Nie Z., Hu G., Wei G. (2012). c-Myc is a universal amplifier of expressed genes in lymphocytes and embryonic stem cells. *Cell*.

[79] Lin C. Y., Lovén J., Rahl P. B. (2012). Transcriptional amplification in tumor cells with elevated c-Myc. *Cell*.

[80] Carey B. W., Markoulaki S., Hanna J. H. (2011). Reprogramming factor stoichiometry influences the epigenetic state and biological properties of induced pluripotent stem cells. *Cell Stem Cell*.

[81] Wang S.-H., Tsai M.-S., Chiang M.-F., Li H. (2003). A novel NK-type homeobox gene, *ENK* (early embryo specific NK), preferentially expressed in embryonic STEM CELLS. *Gene Expression Patterns*.

[82] Chambers I., Colby D., Robertson M. (2003). Functional expression cloning of Nanog, a pluripotency sustaining factor in embryonic stem cells. *Cell*.

[83] Mitsui K., Tokuzawa Y., Itoh H. (2003). The homeoprotein nanog is required for maintenance of pluripotency in mouse epiblast and ES cells. *Cell*.

[84] Hyslop L., Stojkovic M., Armstrong L. (2005). Downregulation of NANOG induces differentiation of human embryonic stem cells to extraembryonic lineages. *STEM CELLS*.

[85] Armstrong L., Hughes O., Yung S. (2006). The role of PI3K/AKT, MAPK/ERK and NF*κβ* signalling in the maintenance of human embryonic stem cell pluripotency and viability highlighted by transcriptional profiling and functional analysis. *Human Molecular Genetics*.

[86] Chen Y., Du Z., Yao Z. (2006). Roles of the Nanog protein in murine F9 embryonal carcinoma cells and their endoderm-differentiated counterparts. *Cell Research*.

[87] Chambers I., Silva J., Colby D. (2007). Nanog safeguards pluripotency and mediates germline development. *Nature*.

[88] Silva J., Chambers I., Pollard S., Smith A. (2006). Nanog promotes transfer of pluripotency after cell fusion. *Nature*.

[89] Silva J., Nichols J., Theunissen T. W. (2009). Nanog is the gateway to the pluripotent ground state. *Cell*.

[90] Festuccia N., Osorno R., Halbritter F. (2012). Esrrb is a direct Nanog target gene that can substitute for Nanog function in pluripotent cells. *Cell Stem Cell*.

[91] Buganim Y., Faddah D. A., Cheng A. W. (2012). Single-cell expression analyses during cellular reprogramming reveal an early stochastic and a late hierarchic phase. *Cell*.

[92] Cox J. L., Mallanna S. K., Luo X., Rizzino A. (2010). Sox2 uses multiple domains to associate with proteins present in Sox2-protein complexes. *PLoS ONE*.

[93] Yuan J., Nguyen C. K., Liu X., Kanellopoulou C., Muljo S. A. (2012). Lin28b reprograms adult bone marrow hematopoietic progenitors to mediate fetal-like lymphopoiesis. *Science*.

[94] Molenaar J. J., Domingo-Fernández R., Ebus M. E. (2012). LIN28B induces neuroblastoma and enhances MYCN levels via let-7 suppression. *Nature Genetics*.

[95] Xu H. M., Liao B., Zhang Q. J. (2004). Wwp2, An E3 ubiquitin ligase that targets transcription factor Oct-4 for ubiquitination. *Journal of Biological Chemistry*.

[96] Saxe J. P., Tomilin A., Schöler H. R., Plath K., Huang J. (2009). Post-translational regulation of Oct4 transcriptional activity. *PLoS ONE*.

[97] Liao B., Jin Y. (2010). Wwp2 mediates Oct4 ubiquitination and its own auto-ubiquitination in a dosage-dependent manner. *Cell Research*.

[98] Pickart C. M., Fushman D. (2004). Polyubiquitin chains: polymeric protein signals. *Current Opinion in Chemical Biology*.

[99] Polo J. M., Anderssen E., Walsh R. M. (2012). A molecular roadmap of reprogramming somatic cells into iPS cells. *Cell*.

[100] Buckley S. M., Aranda-Orgilles B., Strikoudis A. (2012). Regulation of pluripotency and cellular reprogramming by the ubiquitin-proteasome system. *Cell Stem Cell*.

[101] Chen Z. Y., Wang X., Zhou Y., Offner G., Tseng C.-C. (2005). Destabilization of Krüppel-like factor 4 protein in response to serum stimulation involves the ubiquitin-proteasome pathway. *Cancer Research*.

[102] Hu D., Wan Y. (2011). Regulation of Krüppel-like factor 4 by the anaphase promoting complex pathway is involved in TGF-*β* signaling. *The Journal of Biological Chemistry*.

[103] Kim M. O., Kim S.-H., Cho Y.-Y. (2012). ERK1 and ERK2 regulate embryonic stem cell self-renewal through phosphorylation of Klf4. *Nature Structural & Molecular Biology*.

[104] Hann S. R., Eisenman R. N. (1984). Proteins encoded by the human c-myc oncogene: differential expression in neoplastic cells. *Molecular and Cellular Biology*.

[105] Reed S. I. (2003). Ratchets and clocks: the cell cycle, ubiquitylation and protein turnover. *Nature Reviews Molecular Cell Biology*.

[106] Gross-Mesilaty S., Reinstein E., Bercovich B. (1998). Basal and human papillomavirus E6 oncoprotein-induced degradation of Myc proteins by the ubiquitin pathway. *Proceedings of the National Academy of Sciences of the United States of America*.

[107] Salghetti S. E., Kim S. Y., Tansey W. P. (1999). Destruction of Myc by ubiquitin-mediated proteolysis: cancer-associated and transforming mutations stabilize Myc. *The EMBO Journal*.

[108] Gregory M. A., Hann S. R. (2000). c-Myc proteolysis by the ubiquitin-proteasome pathway: stabilization of c-Myc in Burkitt's lymphoma cells. *Molecular and Cellular Biology*.

[109] Hann S. R. (2006). Role of post-translational modifications in regulating c-Myc proteolysis, transcriptional activity and biological function. *Seminars in Cancer Biology*.

[110] Flinn E. M., Busch C. M. C., Wright A. P. H. (1998). myc boxes, which are conserved in myc family proteins, are signals for protein degradation via the proteasome. *Molecular and Cellular Biology*.

[111] Belizario J. E., Alves J., Garay-Malpartida M., Occhiucci J. M. (2008). Coupling caspase cleavage and proteasomal degradation of proteins carrying PEST motif. *Current Protein and Peptide Science*.

[112] Rechsteiner M., Rogers S. W. (1996). PEST sequences and regulation by proteolysis. *Trends in Biochemical Sciences*.

[113] Carafoli E., Molinari M. (1998). Calpain: a protease in search of a function?. *Biochemical and Biophysical Research Communications*.

[114] Small G. W., Chou T.-Y., Dang C. V., Orlowski R. Z. (2002). Evidence for involvement of calpain in c-Myc proteolysis in vivo. *Archives of Biochemistry and Biophysics*.

[115] Kim S. Y., Herbst A., Tworkowski K. A., Salghetti S. E., Tansey W. P. (2003). Skp2 regulates Myc protein stability and activity. *Molecular Cell*.

[116] von der Lehr N., Johansson S., Wu S. (2003). The F-box protein Skp2 participates in c-Myc proteosomal degradation and acts as a cofactor for c-Myc-regulated transcription. *Molecular Cell*.

[117] Welcker M., Orian A., Jin J. (2004). The Fbw7 tumor suppressor regulates glycogen synthase kinase 3 phosphorylation-dependent c-Myc protein degradation. *Proceedings of the National Academy of Sciences of the United States of America*.

[118] Alarcon-Vargas D., Ronai Z. (2004). c-Jun-NH_2_ kinase (JNK) contributes to the regulation of c-Myc protein stability. *Journal of Biological Chemistry*.

[119] Swaney D. L., Wenger C. D., Thomson J. A., Coon J. J. (2009). Human embryonic stem cell phosphoproteome revealed by electron transfer dissociation tandem mass spectrometry. *Proceedings of the National Academy of Sciences of the United States of America*.

[120] Van Hoof D., Muñoz J., Braam S. R. (2009). Phosphorylation dynamics during early differentiation of human embryonic stem cells. *Cell Stem Cell*.

[121] Jeong C.-H., Cho Y.-Y., Kim M.-O. (2010). Phosphorylation of Sox2 cooperates in reprogramming to pluripotent stem cells. *STEM CELLS*.

[122] Ramakrishna S., Suresh B., Lim K.-H. (2011). PEST motif sequence regulating human NANOG for proteasomal degradation. *Stem Cells and Development*.

[123] Moretto-Zita M., Jin H., Shen Z., Zhao T., Briggs S. P., Xu Y. (2010). Phosphorylation stabilizes Nanog by promoting its interaction with Pin1. *Proceedings of the National Academy of Sciences of the United States of America*.

[124] Lim K.-H., Kim S.-R., Ramakrishna S., Baek K.-H. (2014). Critical lysine residues of Klf4 required for protein stabilization and degradation. *Biochemical and Biophysical Research Communications*.

[125] Henry K. W., Wyce A., Lo W.-S. (2003). Transcriptional activation via sequential histone H2B ubiquitylation and deubiquitylation, mediated by SAGA-associated Ubp8. *Genes & Development*.

[126] Köhler A., Zimmerman E., Schneider M., Hurt E., Zheng N. (2010). Structural basis for assembly and activation of the heterotetrameric SAGA histone H2B deubiquitinase module. *Cell*.

[127] Zhang X.-Y., Pfeiffer H. K., Thorne A. W., McMahon S. B. (2008). USP22, an hSAGA subunit and potential cancer stem cell marker, reverses the polycomb-catalyzed ubiquitylation of histone H2A. *Cell Cycle*.

[128] Zhang X.-Y., Varthi M., Sykes S. M. (2008). The putative cancer stem cell marker USP22 is a subunit of the human SAGA complex required for activated transcription and cell-cycle progression. *Molecular Cell*.

[129] Zhao Y., Lang G., Ito S. (2008). A TFTC/STAGA module mediates histone H2A and H2B deubiquitination, coactivates nuclear receptors, and counteracts heterochromatin silencing. *Molecular Cell*.

[130] Glinsky G. V. (2006). Genomic models of metastatic cancer: functional analysis of death-from-cancer signature genes reveals aneuploid, anoikis-resistant, metastasis-enabling phenotype with altered cell cycle control and activated Polycomb Group (PcG) protein chromatin silencing pathway. *Cell Cycle*.

[131] Lin Z., Yang H., Kong Q. (2012). USP22 antagonizes p53 transcriptional activation by deubiquitinating Sirt1 to suppress cell apoptosis and is required for mouse embryonic development. *Molecular Cell*.

[132] Sridharan R., Tchieu J., Mason M. J. (2009). Role of the murine reprogramming factors in the induction of pluripotency. *Cell*.

[133] Sussman R. T., Stanek T. J., Esteso P., Gearhart J. D., Knudsen K. E., McMahon S. B. (2013). The epigenetic modifier ubiquitin-specific protease 22 (USP22) regulates embryonic stem cell differentiation via transcriptional repression of sex-determining region Y-box 2 (SOX2). *The Journal of Biological Chemistry*.

[134] Lander G. C., Estrin E., Matyskiela M. E., Bashore C., Nogales E., Martin A. (2012). Complete subunit architecture of the proteasome regulatory particle. *Nature*.

[135] Boyer L. A., Lee T. I., Cole M. F. (2005). Core transcriptional regulatory circuitry in human embryonic stem cells. *Cell*.

[136] Ramalho-Santos M., Yoon S., Matsuzaki Y., Mulligan R. C., Melton D. A. (2002). ‘Stemness’: transcriptional profiling of embryonic and adult stem cells. *Science*.

[137] Blanpain C., Lowry W. E., Geoghegan A., Polak L., Fuchs E. (2004). Self-renewal, multipotency, and the existence of two cell populations within an epithelial stem cell niche. *Cell*.

[138] Huang Z., Wu Q., Guryanova O. A. (2011). Deubiquitylase HAUSP stabilizes REST and promotes maintenance of neural progenitor cells. *Nature Cell Biology*.

[139] Fuchs G., Shema E., Vesterman R. (2012). RNF20 and USP44 regulate stem cell differentiation by modulating H2B monoubiquitylation. *Molecular Cell*.

